# Patient characteristics associated with hospitalisations for ambulatory care sensitive conditions in Victoria, Australia

**DOI:** 10.1186/1472-6963-12-475

**Published:** 2012-12-21

**Authors:** Zahid Ansari, Syed Imran Haider, Humaira Ansari, Tanyth de Gooyer, Colin Sindall

**Affiliations:** 1 Department of Health, Health Intelligence Unit, Prevention and Population Health, 50 Lonsdale Street, Melbourne, 3000, VIC, Australia; 2 Adjunct Clinical Associate Professor, School of Public Health and Preventive Medicine, Monash University, Melbourne, VIC, Australia; 3 School of Medicine, University of Melbourne, Melbourne, VIC, Australia

**Keywords:** Ambulatory care, Primary care, Socio-demographic, Access barriers

## Abstract

**Background:**

Ambulatory Care Sensitive Conditions (ACSCs) are those for which hospitalisation is thought to be avoidable with the application of preventive care and early disease management, usually delivered in a primary care setting. ACSCs are used extensively as indicators of accessibility and effectiveness of primary health care. We examined the association between patient characteristics and hospitalisation for ACSCs in the adult and paediatric population in Victoria, Australia, 2003/04.

**Methods:**

Hospital admissions data were merged with two area-level socioeconomic indexes: Index of Socio-Economic Disadvantage (IRSED) and Accessibility/Remoteness Index of Australia (ARIA). Univariate and multiple logistic regressions were performed for both adult (age 18+ years) and paediatric (age <18 years) groups, reporting odds ratios (OR) and 95% confidence intervals (CI) for a number of predictors of ACSCs admissions compared to non-ACSCs admissions.

**Results:**

Predictors were much more strongly associated with ACSCs admissions compared to non-ACSCs admissions in the adult group than for the paediatric group with the exception of rurality. Significant adjusted ORs in the adult group were 1.06, 1.15, 1.13, 1.06 and 1.11 for sex, rurality, age, IRSED and ARIA variables, and 1.34, 1.04 and 1.09 in the paediatric group for rurality, IRSED and ARIA, respectively.

**Conclusions:**

Disadvantaged paediatric and adult population experience more need of hospital care for ACSCs. Access barriers to primary care are plausible causes for the observed disparities. Understanding the characteristics of individuals experiencing access barriers to primary care will be useful for developing targeted interventions meeting the unique ambulatory needs of the population.

## Background

Ambulatory Care Sensitive Conditions (ACSCs) are those for which hospitalisation is thought to be avoidable with the application of preventive care and early disease management, usually delivered in an primary care setting [1-4]. In theory, timely and effective ambulatory care can reduce the risks of hospitalisation by preventing the onset of an illness or condition; controlling an acute episodic illness or condition; or managing a chronic disease condition [1-4]. A broad range of conditions have been identified as ambulatory care sensitive in the literature [2-6].

ACSCs have been used extensively as an indicator of accessibility and overall effectiveness of primary health care, such that the rate of hospitalisation for ACSCs is higher in communities with poor access to ambulatory care [2,3,7-9]. Used as an indicator, ACSCs are of interest to policy makers in public health and health services research as they represent the occurrence of avoidable morbidity, providing an evidence-based foundation for targeted interventions to control costs of health care provided at public expense [10-20].

Factors that have previously been examined to explain reasons for differentials in ACSCs admission rates across geographic areas and subgroups of populations include: demographic, socio-economic status (SES), rurality, health system factors, prevalence, life style factors, environment, adherence to medication, propensity to seek care, and severity of illness [21].

ACSCs admission rates have been stratified by age and sex in several studies. Hospitalisation rates for ACSCs are steady in the age groups 19-64 years, with higher rates of admissions in younger (<=18 years) and older (>=65 years) age groups [22]. Older age groups (65+) have been studied extensively in the US, with significant increases in the rates of ACSCs admissions in this age category between 1980 and 1998 [23].

Studies examining SES and ACSCs hospitalisations have also been conducted [2,4,6,8,9,17,18,20,24-35]. Most of these studies found that at least one SES variable (such as income, education, occupation and insurance status) was a statistically significant strong predictor of ACSCs hospitalisation, independently of other measured variables.

Many studies have analysed the effect of location of patients’ residence on ACSCs admission rates [17,20,26,27,32,34-39]. These studies consistently found that the highest rates of hospitalisations for ACSCs admissions occurred in the most rural areas compared to their respective counterparts residing in metropolitan areas.

Only a few studies have considered the joint impact of demographic and socioeconomic characteristics on ACSCs hospitalisations, or examined the different pattern which might pertain to paediatric and adult hospitalisations. From a policy perspective, it is important to understand the profiles of individuals experiencing access barriers to primary care as it can assist in the development of policies and programs that improve access for those individuals [27]. Victoria is the second largest state in Australia with a population of 4.8 million people in 2003. Examining the characteristics of patients may be useful in the development of policies and programs to improve access to primary health care in the state of Victoria.

The aim of this study was to examine the association between patient characteristics and hospitalisation for ACSCs in the adult and paediatric population groups in Victoria, Australia, 2003/04. More specifically, this paper reports on differentials in ASCSs admission rates among subgroups of the population included SES and rurality.

## Methods

Data were obtained at both the individual- and aggregate (ecologic) level.

### Individual-level data

Hospital separation data were obtained from the 2003/04 Victorian Admitted Episodes Dataset (VAED) [40]. The VAED is a minimum dataset containing demographic, clinical and administrative data for every admitted episode of care occurring in all Victorian acute hospitals, both public and private. Clinical data are stored as ICD-10-AM codes in 40 diagnosis and procedure fields in the VAED [41].

The ACSCs identified using the ICD-10-AM codes were based on the published literature [2-4]. The VAED records were selected based on diagnosis fields and some exclusions were made based on procedure fields (Table 1). Binary dependent variables were created based on the presence or absence of ACSCs conditions (separate and overall). The definitions of the ACSCs diagnostic categories used in this study are set out in Table 1. Most of the 19 ACSCs are based on principal diagnosis, while 3 (influenza/pneumonia, other vaccine preventable and diabetes complications) are based on any diagnosis field (not necessarily the reason for admission). The argument for this is that any instance of, for example, vaccine preventable conditions or diabetes complications clearly indicates a failure to access timely primary care, irrespective of reason for admission. This is because the occurrence of even a single such case, even in the community, should not happen given the ideal functioning of the primary care system (in the delivery of immunisation services and diabetes management services in these cases) and the existence of such cases can be useful as indicators of lack of optimal primary care. These conditions stand in contrast to the others, for example asthma, where clearly a secondary (not principal) diagnosis of asthma (perhaps mild and well-managed) in no way can be seen as reflecting adversely on the primary care system, whereas a case of asthma causing an admission can be seen as an indicator of failure of primary care to manage the condition satisfactorily. All these definitions and codes have been part of the Victorian ACSCs study and widely published in Australia and internationally [2-4,7,11,42-44].

**Table 1:** ICD-10-AM codes used to identify Ambulatory Care Sensitive Conditions (ACSCs) and exclusions made based on procedure fields

**Category**	**ICD10 codes**	**Notes (ICD10)**
Influenza and pneumonia	J10 J11 J13 J14 J153 J154 J157 J159 J168 J181 J188	In any diagnosis field, excludes cases with secondary diagnosis of D57, and people under 2 months
Other vaccine preventable	A35 A36 A37 A80 B05 B06 B161 B169 B180 B181 B26 G000 M014	In any diagnosis field
Asthma	J45 J46	Principal diagnosis only
Congestive heart failure*	I50 I110 J81	Principal diagnosis only, exclude cases with procedure codes according to attached list
Diabetes complications	E101 E102 E103 E104 E105 E106 E107 E108 E110 E111 E112 E113 E114 E115 E116 E117 E118 E130 E131 E132 E133 E134 E135 E136 E137 E138 E140 E141 E142 E143 E144 E145 E146 E147 E148	In any diagnosis field
Chronic obstructive pulmonary disease	J20 J41 J42 J43 J44 J47	Principal diagnosis only, J20 only with diag2 of J41 J42 J43 J47 J44
Angina	I20 I240 I248 I249	Principal diagnosis only, exclude cases with procedure codes NOT in blocks 1820 to 2140
Iron deficiency anaemia	D501 D508 D509	Principal diagnosis only
Hypertension*	I10 I119	Principal diagnosis only, exclude cases with procedure codes according to attached list
Nutritional deficiencies	E40 E41 E42 E43 E550 E643	Principal diagnosis only
Dehydration and gastroenteritis	E86 K522 K528 K529	Principal diagnosis only
Pyelonephritis	N390 N10 N12 N11 N136	Principal diagnosis only
Perforated/bleeding ulcer	K250 K251 K252 K254 K255 K256 K260 K261 K262 K264 K265 K266 K270 K271 K272 K274 K275 K276 K280 K281 K282 K284 K285 K286	Principal diagnosis only
Cellulitis	L03 L04 L08 L980 L88 L983	Principal diagnosis only, exclude cases with any procedure except those in blocks 1820 to 2016 or if procedure is 30216-02 30676-00 30223-02 30064-00 34527-01 34527-00 90661-00 and this is the only listed procedure
Pelvic inflammatory disease	N70 N73 N74	Principal diagnosis only
Ear, nose and throat infections	H66 H67 J02 J03 J06 J312	Principal diagnosis only
Dental conditions	K02 K03 K04 K05 K06 K08 K098 K099 K12 K13	Principal diagnosis only
Convulsions and epilepsy	O15 G40 G41 R56	Principal diagnosis only
Gangrene	R02	In any diagnosis field

*Procedure codes to use for exclusions for congestive heart failure and hypertension:

33172-00, 35304-00, 35305-00, 35310-02, 35310-00, 38281-11, 38281-07, 38278-01.

38278-00, 38281-02, 38281-01, 38281-00, 38256-00, 38278-03, 38284-00, 38284-02.

38521-09, 38270-01, 38456-19, 38456-15, 38456-12, 38456-11, 38456-10, 38456-07.

38456-01, 38470-00, 38475-00, 38480-02, 38480-01, 38480-00, 38488-06, 38488-04.

38489-04, 38488-02, 38489-03, 38487-00, 38489-02, 38488-00, 38489-00, 38490-00.

38493-00, 38497-04, 38497-03, 38497-02, 38497-01, 38497-00, 38500-00, 38503-00.

38505-00, 38521-04, 38606-00, 38612-00, 38615-00, 38653-00, 38700-02, 38700-00.

38739-00, 38742-02, 38742-00, 38745-00, 38751-02, 38751-00, 38757-02, 38757-01.

38757-00, 90204-00, 90205-00, 90219-00, 90224-00.

Variables used in the analysis at the individual level were age and sex. Age was used to classify episodes into two groups: adult (age 18 years or over) and paediatric (age under 18 years) and was also used as a continuous measure for the regression analyses; adult and paediatric groups were analysed separately.

### Aggregate-level data

The Australian Standard Geographic Classification (ASGC) developed and maintained by the Australian Bureau of Statistics (ABS) defines the boundaries of Local Government Areas (LGA) of which there were 79 in Victoria in the study period [45]. Each individual record was assigned a LGA code.

### Index of relative socioeconomic disadvantage (IRSED)

The ABS has compiled Socioeconomic Indexes for Areas (SEIFA) from individual census variables aggregated at the local area level [45]. SEIFA are measures of the social and economic status of individuals derived from a principal components analysis of variables from the 1996 Australian Census of Population and Housing summarised at a local area level. The SEIFA indices have been standardised to have a mean of 1000.0 across all Census Collection Districts with a standard deviation of 100.0. We have selected one of these indexes, IRSED, for use in this study.

The IRSED is derived from summing multiple weighted variables including education, occupation, non-English speaking background, Indigenous origin, and the economic resources of households. The higher the value of IRSED, the less disadvantaged the area is compared to others. Average IRSED scores were calculated for each LGA and ranked from highest to lowest. LGAs were then assigned to one of five quintiles of approximately equal population from Q1 (highest socioeconomic disadvantage) to Q5 (lowest socioeconomic disadvantage).

### Accessibility/remoteness index of Australia (ARIA)

ARIA provides a measure of the relative accessibility and remoteness of Victorian local areas for the study [46]. ARIA uses a database of road, locality and service information to provide an objective measure of remoteness (defined as lack of accessibility to services regarded as “normal” in urban areas). When applied to Victorian statistical local areas (SLAs), this index varies from less than 1.84 (highly accessible – relatively unrestricted accessibility to a wide range of goods and services) to between 3.51 and 5.80 (moderately accessible – significantly restricted accessibility of goods, services and opportunities for social interaction). LGAs were assigned to one of three levels of accessibility according to average ARIA score:

1. Highly Accessible (ARIA score 0 - 1.84) - relatively unrestricted accessibility to a wide range of goods and services and opportunities for social interaction.

2. Accessible (ARIA score >1.84 - 3.51) - some restrictions to accessibility of some goods, services and opportunities for social interaction.

3. Moderately Accessible (ARIA score >3.51-5.80) - significantly restricted accessibility of goods, services and opportunities for social interaction.

### Rural/metropolitan variable

An additional variable directly derived from SLAs was rural/metropolitan location based on the eight Department of Health Regions in Victoria (three metropolitan and five rural) with boundaries aligned with LGAs.

### Coding of variables

We first selected all records from the VAED with separation dates in the period 1 July 2003 to 30 June 2004 and where usual residence of the patient was a Victorian LGA. We then constructed 20 binary variables (one for each of the 19 ACSCs and one overall ACSC variable for which ACSC = 1 if the episode was associated with any ACSC condition and ACSC = 0 otherwise).

We then selected all records with ACSC = 1 and made a random selection from all the remaining records with ACSC = 0, so that the number of records in the two groups were equal.

### Data analysis

STATA software was used to perform logistic regression with either: 1) a single dependent variable and a single independent variable (simple logistic regression) reporting unadjusted Odds Ratios (OR) and 95% confidence intervals (CI); or 2) with a single dependent variable and multiple independent variables (multiple logistic regression) reporting adjusted OR and 95% CI. Separate logistic regressions were performed for both adult (age 18+) and paediatric (age <18) groups.

Simple logistic regressions were performed using ACSC (0/1) as the dependent variable and for each independent variable separately in turn. Then a multiple logistic regression was performed using ACSC (0/1) as the dependent variable.

Records for which ACSC = 1 were then selected and the top five conditions (sorted by number of admissions) for the adult and paediatric groups separately were chosen to be used as the dependent binary variable for simple logistic regressions e.g. DIAB = 1 if the diabetes complications ACSC was associated with an episode and DIAB = 0 otherwise.

For all analyses, OR and 95% CI were reported. For the binary variables the reference category is stated (OR=1) and for the nominal scale variables (treated as continuous) the odds ratios for an increase (or decrease) of one category unit is reported.

As the data had individual and aggregate level variables, we used random effect multi-level generalised linear models to identify the influence of these variables on ACSCs [47]. These models were fitted using the command GLLAMMs (Generalised Linear Latent And Mixed Models) in STATA [48,49]. These models took into account the nesting of subjects within LGAs and provided robust standard errors.

## Results

### Study cohort

The study cohort consisted of all individuals admitted as inpatients to any hospital (public or private) in the state of Victoria, Australia and discharged in the period 1 July 2003 to 30 June 2004 who had one of the ACSCs listed in Table 1. In addition, a contrast group was assembled consisting of a random sample of patients who did not have an ACSC. 47.9% of the patients were males and 52.1% were females. 11.0% of patients were paediatric patients. A lower proportion (29.6%) of patients lived in rural areas compared to metropolitan areas. 3.3% of patients lived in ARIA 3 (lowest accessibility) and 19.6% of patients belonged to Q1 (highest socioeconomic disadvantage). Details of crude (unadjusted) hospitalisation rates by age-groups and sex are reported in Table 2. The paediatric group of patients had admission rates tracking a similar age profile between the ACSCs and non-ACSCs cohorts, while the adult ACSCs cohort had lower rates than the non-ACSCs for ages 18-64, and higher rates for age 65+.

**Table 2:** Ambulatory Care Sensitive Conditions (ACSCs) and non-ACSCs hospitalisation rate in the adult and paediatric population in Victoria, 2003/04

	**Hospitalisations per 1,000 population**			
**Age group**	**Adult ACSCs**	Adult Non-ACSCs^*^	**Paediatric*ACSCs***	Paediatric Non-ACSCs^*^
00-05			34.60	29.70
06-17			10.12	7.97
18-24	12.29	18.95		
25-34	12.89	27.68		
35-44	15.54	27.22		
45-54	23.45	33.59		
55-64	46.00	50.22		
65-74	92.01	77.91		
75-84	168.05	103.62		
85+	206.03	96.04		
Males	40.57	38.05	18.79	15.75
Females	39.79	43.99	16.95	13.94

^*^The numerator for non-ACSCs was taken to be the sample from the Victorian Admitted Episode Dataset (VAED) to match the numbers of ACSCs rather than all non-ACSCs admissions for 2003-04. Hence non-ACSCs admission rates are an underestimate of the true rates of non-ACSCs admissions in the population in 2003-04.

### Total ACSCs admissions

There were a total of 171 782 admissions for ACSCs (151 114 adult; 20 668 paediatric). The crude admission rates for both ACSCs and non-ACSCs increased with age among the adult population, and decreased with age among the paediatric group (Table 2). The significant predictors of ACSCs admissions in Victoria 2003/04 varied between the adult and paediatric groups. For both the adult and paediatric groups, rurality and area of residence of least accessibility were significantly associated with total ACSCs admissions (Table 3).

**Table 3:** Predictors of Ambulatory Care Sensitive Conditions (ACSCs) admissions compared to non-ACSCs admissions for adult and paediatric admissions in Victoria (2003-04)

		**Adult**			**Paediatric**		
**Predictor**	**Reference (Binary variable)**	**Unadjusted OR*(LCI, UCI)***	**Adjusted OR*(LCI, UCI)***	**Direction of increased likelihood of ACSC (p<0.05)**	**Unadjusted OR*(LCI, UCI)***	**Adjusted OR*(LCI, UCI)***	**Direction ofincreased likelihood of ACSC (p<0.05)**
Male/Female	Female	1.18 *(1.13, 1.23)*	1.06 *(1.02, 1.10)*	Male	0.98 *(0.92, 1.04)*	0.99 *(0.93, 1.05)*	—
Rural/Metro	Metro	1.45 *(1.24, 1.70)*	1.15 *(1.05, 1.27)*	Rural	1.40 *(1.27, 1.54)*	1.34 *(1.22, 1.48)*	Rural
Increase in OR of ACSC per 5 yr. increase in Age	*Continuous variable*	1.12 *(1.12, 1.13)*	1.13 *(1.12, 1.14)*	Older	0.95 *(0.94, 0.97)*	0.95 *(0.93, 0.97)*	Younger
Increase in OR of ACSC per decrease in IRSED quintile	*Continuous variable*	1.08 *(1.03, 1.14)*	1.06 *(1.04, 1.08)*	IRSED Q1 (greater disadvantage)	1.03 *(0.98, 1.08)*	1.04 *(1.00, 1.08)*	—
Increase in OR of ACSC per increase in ARIA category	*Continuous variable*	1.39 *(1.29, 1.50)*	1.11 *(1.02, 1.21)*	ARIA 3 (less accessible)	1.26 *(1.15, 1.38)*	1.09 *(1.01, 1.19)*	ARIA 3 (less accessible)

Data are presented as unadjusted (univariable) and adjusted (multivariable) odds ratios (OR) and 95% lower and upper confidence intervals (LCI, UCI).

For the adult population, significant differences in ACSCs admission rates were observed across sex, age, place of residence, degree of remoteness, and socio-economic disadvantage (Table 3):

i. Males were 6% more likely to be admitted with ACSCs admissions compared to females;

ii. ACSCs admission rates increased by 13% per five-year increase in age category;

iii. ACSCs admissions were 15% more likely among rural residents compared to their metropolitan counterpart;

iv. ACSCs admission rates increased by 11% for each degree increase in ARIA category; and

v. Increasing socio-economic disadvantage of an area was associated with 6% increase in ACSCs admission rates.

In the paediatric group, significant predictors included rural residence, degree of remoteness and younger age. The unadjusted and adjusted odds ratios were almost always in the same direction (above or below 1.0). The adjusted ORs tended to lean more towards the null, although the unadjusted and adjusted ORs were either both significant (p<0.05) or neither was significant. Predictors were much more strongly associated with ACSCs admissions in the adult (18+ years) group than for the paediatric (<18) group, with the exception of rurality (adjusted OR = 1.15 for the adult group, adjusted OR = 1.34 in the paediatric group).

### Specific ACSCs admissions

The top five adult ACSCs admissions and number of episodes in 2003/04 were: diabetes complications (N = 55 007), chronic obstructive pulmonary disease (N = 13 555), dehydration and gastroenteritis (N = 12 145), congestive heart failure (N = 11 676) and angina (N = 11 130).

Conditional on admission for ACSCs, the characteristics of the adult group that were significantly (p<0.05) associated with each of the top five ACSCs admissions (shown in Tables 4, 5, 6) were:

1. Diabetes complications Male, ARIA 3 (lowest accessibility), IRSED quintile (linear trend towards high disadvantage), older age group

2. Chronic obstructive pulmonary disease (COPD), Male and older age group;

3. Dehydration and gastroenteritis Female, ARIA 1-2 (not the lowest accessibility), younger age group, IRSED quintile (linear trend towards low disadvantage);

4. Congestive heart failure ARIA 1-2 (not the lowest accessibility), Older age group;

5. Angina Rural residence, male, ARIA 2-3 (not the highest accessibility), older age group.

**Table 4:** Top 5 Adult and paediatric individual Ambulatory Care Sensitive Conditions (ACSCs) compared to total ACSCs by gender and locality in Victoria (2003-04)

	**Male**	**Female*(Reference)***	OR*(LCI, UCI)	Predictor direction* (p<0.05)	**Rural**	**Metro*(Reference)***	OR*(LCI, UCI)	Predictor direction* (p<0.05)
Adult								
Diabetes complications	30,861	24,146	1.57 *(1.44, 1.71)*	Male	19,294	35,713	1.13 *(0.90, 1.40)*	—
COPD	7,428	6,127	1.29 *(1.16, 1.42)*	Male	4,680	8,875	1.06 *(0.95, 1.17)*	—
Dehydration & gastroenteritis	4,686	7,459	0.63 *(0.58, 0.68)*	Female	3,726	8,419	0.87 *(0.72, 1.05)*	—
Congestive heart failure	5,662	6,014	0.97 *(0.91, 1.04)*	—	3,710	7,966	0.92 *(0.80, 1.06)*	—
Angina	4,934	6,196	1.33 *(1.22, 1.45)*	Male	4,525	6,605	1.40 *(1.20, 1.63)*	Rural
Total ACSCs	72,149	74,794	1.00 *(Reference)*	—	49,118	97,825	1.00 *(Reference)*	—
Paediatric								
Dental Conditions	2,987	2,718	0.92 *(0.86, 0.98)*	Female	2,561	3,144	1.68 *(1.33, 2.13)*	Rural
Asthma	2,845	1,797	1.49 *(1.39, 1.59)*	Male	1,345	3,297	0.66 *(0.55, 0.79)*	Metro
Ear, nose & throat infection	2,216	1,861	1.03 *(0.96, 1.10)*	—	1,530	2,547	1.08 *(0.84, 1.38)*	—
Convulsions & epilepsy	1,164	961	1.04 *(0.94, 1.16)*	—	774	1,351	1.02 *(0.80, 1.29)*	—
Pyelonephritis	382	768	**0.41*(0.34, 0.48)***	**Female**	291	859	0.58 *(0.50, 0.68)*	Metro
Total ACSCs	10,860	9,321	1.00 *(Reference)*	—	7,288	12,893	1.00 *(Reference)*	—

Data are presented as univariable odds ratios (OR) and 95% lower and upper confidence intervals (LCI, UCI) for gender and locality predictors.

* **Bold** indicates OR < 0.50.

**Table 5:** Top 5 Adult and paediatric individual Ambulatory Care Sensitive Conditions (ACSCs) in Victoria (2003-04) compared to total ACSCs by Accessibility Remoteness Index of Australia (ARIA) categories

	**ARIA 3 (Less Accessible)**	**ARIA 1-2*(Reference)***	OR*(LCI, UCI)	Predictor direction* (p<0.05)	**ARIA 1 (Highly Accessible)**	**ARIA 2-3*(Reference)***	OR*(LCI, UCI)	Predictor direction* (p<0.05)
Adult								
Diabetes complications	2,901	52,106	1.54 *(1.00, 2.37)*	—	47,939	7,068	0.83 *(0.62, 1.11)*	—
COPD	468	13,087	0.81 *(0.61, 1.07)*	—	11,973	1,582	1.00 *(0.85, 1.17)*	**—**
Dehydration & gastroenteritis	373	11,772	0.71 *(0.58, 0.88)*	ARIA 1-2	10,838	1,307	1.10 *(0.88, 1.37)*	—
Congestive heart failure	381	11,295	0.76 *(0.63, 0.92)*	ARIA 1-2	10,349	1,327	1.03 *(0.86, 1.24)*	—
Angina	482	10,648	1.05 *(0.82, 1.34)*	—	9,608	1,522	0.82 *(0.69, 0.97)*	ARIA2-3
Total ACSCs	6,103	140,840	1.00 *(Reference)*	—	129,842	17,101	1.00 *(Reference)*	—
Paediatric								
Dental Conditions	425	5,280	**2.53*(2.01, 3.18)***	**ARIA 3**	4,845	860	0.66 *(0.47, 0.94)*	ARIA2-3
Asthma	124	4,518	**0.38*(0.29, 0.78****)*	**ARIA 1-2**	4,181	461	1.28 *(0.95, 1.71)*	—
Ear, nose & throat infection	162	3,915	0.90 *(0.75, 1.08)*	—	3,533	544	0.84 *(0.66, 1.06)*	—
Convulsions & epilepsy	56	2,069	0.57 *(0.46, 0.71)*	ARIA 1-2	1,937	188	1.42 *(1.18, 1.72)*	ARIA 1
Pyelonephritis	21	1,129	**0.40*(0.27, 0.58)***	**ARIA 1-2**	1,067	83	1.76 *(1.34, 2.32)*	ARIA 1
Total ACSCs	872	19,309	1.00 *(Reference)*	—	17,801	2,380	1.00 *(Reference)*	—

Data are presented as univariable odds ratios (OR) and 95% lower and upper confidence intervals (LCI, UCI) by predictors (ARIA 3 vs ARIA 1-2, and ARIA 1 vs ARIA 2-3).

* **Bold** indicates OR > 2.00 or OR < 0.50.

**Table 6:** Top 5 Adult and paediatric individual Ambulatory Care Sensitive Conditions (ACSCs) in Victoria (2003-04) compared to total ACSCs by age and Index of Relative Socio-economic Disadvantage (IRSED)

	**N =**	**OR*(LCI, UCI)*per 5 year increase in Age**	**Predictor direction (p<0.05)**	**N =**	**OR*(LCI, UCI)*per decrease in each IRSED category (1-5)**	**Predictor direction (p<0.05)**
Adult						
Diabetes complications	55,007	1.10 *(1.08, 1.12)*	Older	55,007	1.14 *(1.08, 1.20)*	Q1 (high disadvantage))
COPD	13,555	1.20 *(1.18, 1.21)*	Older	13,555	1.00 *(0.96, 1.03)*	**—**
Dehydration & gastroenteritis	12,145	0.88 *(0.87, 0.89)*	Younger	12,145	0.91 *(0.85, 0.97)*	Q5 (low disadvantage)
Congestive heart failure	11,676	1.45 *(1.41, 1.48)*	Older	11,676	0.95 *(0.90, 1.00)*	—
Angina	11,130	1.10 *(1.09, 1.11)*	Older	11,130	1.03 *(0.96, 1.10)*	—
Paediatric						
Dental Conditions	5,705	1.23 *(1.16, 1.31)*	Older	5,705	1.01 *(0.92, 1.11)*	—
Asthma	4,642	0.83 *(0.80, 0.88)*	Younger	4,642	1.08 *(0.92, 1.17)*	—
Ear, nose & throat infection	4,077	0.62 *(0.57, 0.67)*	Younger	4,077	0.92 *(0.86, 0.98)*	Q5 (low disadvantage)
Convulsions & epilepsy	2,125	0.89 *(0.82, 0.98)*	Younger	2,125	0.97 *(0.87, 1.08)*	—
Pyelonephritis	1,150	0.80 *(0.68, 0.93)*	Younger	1,150	1.06 *(1.00, 1.14NN*	—

Data are presented as univariable odds ratios (OR) and 95% lower and upper confidence intervals (LCI, UCI) per 5 year increase in age and per decrease inn each IRSED category.

The top five paediatric ACSCs admissions and number of episodes in 2003-04 were: dental conditions (N = 5,705), asthma (N = 4,642), ear, nose and throat infection (N = 4,077), convulsions and epilepsy (N = 2,125) and pyelonephritis (N = 1,150).

Conditional on admission for ACSCs, the characteristics of the paediatric group that were associated with each of the top five ACSCs admissions (shown in Tables 4, 5, 6) were:

1. Dental conditions Female, ARIA 3 (lowest accessibility), rural residence, ARIA 2-3 (not the highest accessibility), older age group;

2. Asthma Male, ARIA 1-2(not the lowest accessibility), metropolitan residence, younger age group;

3. Ear, nose and throat infection Younger age group, IRSED quintile (linear trend towards low disadvantage);

4. Convulsions and epilepsy ARIA 1-2 (not the lowest accessibility), ARIA 1 (highest accessibility), younger age group;

5. Pyelonephritis Female, ARIA 1-2 (not the lowest accessibility), ARIA 1 (highest accessibility), metropolitan residence, younger age group.

## Discussion

The study adds to knowledge of how ACSCs admission rates vary with demographic and socio-economic characteristics in several ways:

1. Unlike previous studies, adult (age>=65) and paediatric (age<=18) admissions are analysed separately. A completely different set of Top 5 (by number of admissions) ACSCs are reported for each age cohort and the associations with demographic and socioeconomic characteristics reported (adjusted and unadjusted for covariates) are also somewhat different.

2. The strength of the associations (adjusted and unadjusted ORs) with demographic and socioeconomic characteristics are reported for ten separate ACSCs diagnostic groups (the Top 5 conditions for each of the Adult and Paediatric cohorts). These results show interesting variations between the individual conditions and with total ACSCs.

3. The approaches to data analysis:

i. A random sample of non-ACSCs admissions was extracted from the VAED data set and compared to the ACSCs admissions to provide estimates of the extent of the associations of ACSCs hospitalisations with demographic and socioeconomic characteristics. The sample size for the non-ACSCs admissions was chosen to be equal to the number of ACSCs admissions. Such a 1:1 ratio is the most efficient for statistical hypothesis testing (narrowest width confidence intervals) relative to the total number of admissions to be analysed. The reason for choosing the non-ACSCs admissions as the reference group for OR calculations was to control for any possible “accessibility to hospital” and/or “propensity to admit” effects likely to be associated with many of the demographic and socioeconomic characteristics used in the study. This technique allowed the ORs in Table 3 to be purer indicators of “access to primary care” alone, without possible contamination by variations in “access to hospital care” per se, since non-ACSCs are not thought to be significantly influenced by access to primary care issues, while ACSCs are.

ii. Individual and contextual variables analysed for the paediatric and adult population using multi-level models.

This study identified significant patient socio-demographic characteristics that were associated with higher admission rates of ACSCs, identifying potential access to primary health care problems faced by the Victorians. In the adult population, the key factors include male sex, older age, rural residence, degree of remoteness, and socio-economic disadvantage. Higher rates of ACSCs admissions in rural areas have been observed in other studies [9,17,20,26,27,36,38,39]. In Victoria, significant differentials between rural and metropolitan areas have been previously described [43]. Key factors *within* rural areas that may be associated with higher ACSCs admission rates include degree of remoteness and accessibility to services, lower number of general practitioners per population suggesting that remote areas have fewer sources of easy to reach primary care, and socio-economic disadvantage [43]. SES has been identified in several studies as important predictor of ACSCs admission rates. This has been observed using individual level measures such as insurance status or access to Medicaid, [4,26,27,50] as well as area level measures, [2,6,8,17,18,24] which were used in this study.

The strength of the study was the analyses of characteristics of both the adult and paediatric population, highlighting potential access barriers faced in both population groups. In both the adult and paediatric groups, rural areas and areas of residence with least accessibility were associated with higher ACSCs admission rates. Male adults were more likely to be admitted with ACSCs admissions, while no significant sex differentials were observed for paediatric groups. In addition, older adults and younger children were more likely to be admitted for ACSCs than younger adults and older children, similar to the findings reported in an earlier study [27]. The findings from our study indicate that male adults, older adults, and younger children may potentially face greater access barriers than female adults and younger adults and older children.

Diabetes complications and dental conditions were the leading cause of hospital admissions in the adult and paediatric populations, respectively. Earlier studies in Victoria have also highlighted significant differentials across communities for diabetes complications, with admission rates significantly higher in rural areas compared to metropolitan [42,51]. There are effective treatments available to prevent the progression of diabetes-related complications. Since these interventions generally achieve the best results if started in the early asymptomatic stages, and complications can progress to an advanced stage before symptoms develop, regular medical screening is essential to identify people who require treatment for their complications [52]. Strategic approaches to manage diabetes have now been developed [53]. Higher admission rates of dental conditions among children, especially in rural areas have also been reported in Victoria [54]. In Victoria, the importance of management of dental conditions in the community has been identified, and strategic approaches to improve the management of dental conditions are being implemented [55].

This study is useful in several ways:

(i) it provides, for the first time in Victoria, simultaneous analyses of adults and paediatric ACSCs. Previous analyses conducted as part of the Victorian ACSCs study [1,7,11,43,56], has not distinguished between adult and paediatric populations;

(ii) it analyses individual and area level characteristics across Victoria using multilevel modelling techniques. Previous analyses of this nature was limited to within rural areas of Victoria [43];

(iii) it identifies potential barriers experienced by the paediatric and adult population in accessing primary care in the community. These barriers can be further explored to identify specific factors that determine if a person is able to access primary health care in a timely and effective manner. Using regularly collected hospital discharge data at the community level, providers and decision makers can easily undertake timely assessments of population needs and the extent of access barriers faced by special population groups [27,28];

(iv) it provides baseline information on ACSCs admission rates for total ACSCs and specific ACSCs in adult and paediatric population. ACSCs admission rates can serve as an information tool for planners and policy makers for continuous monitoring of health services through their inclusion in surveillance system for monitoring acute and chronic conditions in adult and paediatric populations. Changes in ACSCs admissions indicators over time can help pinpoint gaps in the health system providing opportunities for targeted public health and health services interventions; and

(v) through the analyses of hospitalisations for ACSCs, it serves as a convenient and effective evaluation tool to assess the effectiveness of interventions aimed at improving access to primary care. For example, several clinical trials have examined the possibility that better access might reduce ACSCs. One found that greater access after hospital discharge increased rehospitalisation [57]. Other interventions have reduced hospital readmissions for ACSCs through multidisciplinary disease-management teams [58] or advanced discharge planning and home care [59-61]. These clinical studies, for the most part, have focused on seriously ill individuals after hospital discharge. They may not represent the prevention possibilities more generally representative of ACSCs, which have not been widely studied. Given the current wide use of the ACSCs indicator by researchers and policy makers, it would be useful to expand research examining its validity.

(vi) through the analyses of ACSCs indicators, it can contribute to improving efficiency of the health system by identifying opportunities to substitute less expensive model of care. Reducing hospital admissions for ACSCs through better access to primary care in vulnerable subgroups of population not only improves population health, but is also likely to be more cost efficient [27]. Although the cost of improving primary care access is likely to be substantial, most invariably expenditure at outpatient level is still less than that in a hospital setting [27].

The ACSCs in this study included conditions that can be prevented through vaccination e.g., measles, mumps, rubella, tetanus, influenza, and bacterial pneumonia (vaccine preventable ACSCs), acute conditions for which hospitalisations is commonly avoidable with medical interventions available in primary care or the use of antibiotics e.g. dehydration and gastroenteritis, kidney infection, cellulitis, perforated ulcer, ear, nose, and throat infections, pelvic inflammatory disease, and dental conditions (acute ACSCs), and selected chronic conditions that can be managed by life style factors, patient education and pharmaceuticals e.g., diabetes complications, angina, hypertension, asthma, COPD and CCF (chronic ACSCs). Most codes used in this study are available in earlier reports and papers published from the USA and England [4,35,44,62,63]. These codes of ACSCs have been validated as markers of access to primary health care in the USA and Australia [7,8]. However, due to consistent variations in ACSCs definitions and codes, comparison across different datasets and geographic areas is a problem for informing policy and planning [44]. Caminal and Colleagues have recommended that choice of ACSCs should be country specific due to variations in health system between different countries [64].

Access barriers identified in this study are not unique to Victoria. Several international studies have identified access barriers, especially in the disadvantaged populations [7,34,65,66]. For policy makers across the world, the question of access is inextricably linked with equity, one of the key performance indicators of the health system [67]. Most health systems offer inequitable access, and deliver inequitable treatment and outcomes. The goal of equity has not been achieved as seen by significant health differentials between racial, ethnic and socio-economic groups; less than adequate health care provided to vulnerable groups; and policy makers steering away rather than tackling these issues with strong policies.

A recent international survey of individuals’ views of primary health care found that a majority of Australians had been with the same doctor or place of care for more than 5 years and received appointments the same day the last time they needed medical attention [68]. On the other hand, a majority also indicated that accessing primary care after hours was difficult, although the problem in Australia was less widespread than in the United States [68]. Seventeen percent of Australians reported that they did not get medical care because of the cost of a doctor’s visit in the previous 12 months, compared with 6 percent in Canada, 28 percent in New Zealand, 4 percent in the United Kingdom, and 29 percent in the United States [68]. Similar percentages were reported for having skipped a medical test, treatment, or follow-up because of cost [68]. Australians’ reported access barriers are notably greater than those of people in the United Kingdom but also notably less than those of people in the United States. Because of universal insurance in Australia, results of this study may not directly apply to the United States. However, about 43 million Americans are beneficiaries of Medicare, a universal insurance plan of long standing. The prevalence of ACSCs is much higher in the population age 65 and older than in younger populations, as is the occurrence of ACSCs. Thus, the results from Australia may be relevant to the United States’ Medicare system.

In a system that seeks to be egalitarian, equity is the most difficult criteria to operationalise [67]. In this context, it is important to understand that access is multifaceted, not only measuring characteristics of the health system but also characteristics of individuals and the areas it serves [67]. The multiple dimensions of access reflect the need for a new research agenda that includes an expanded primary care and health services research and policy agenda, with a focus on factors that lie outside the health system [67].

Our study is subject to various caveats. The data for this study is from 2003/04. However, more recent ACSCs data from Victoria [69], as well as other jurisdictions [70] and overseas [30,71], indicates that associations with demographic and socioeconomic factors are robust and persist across time. The findings from this study are likely to be still of relevance now, and elsewhere*.* As administrative database was used to identify ACSCs, the recorded diagnoses are prone to coding errors. Reliability and validity of the data in the VAED has been well documented in earlier studies, and is unlikely to have biased our results [72-74]. The 2001 ABS Census of population and Housing was the source of aggregate level data used in this study. The ABS Census of population and Housing is a rich source of data for understanding the socio-demographic makeup, range and variation between communities, with information available down to a small aggregated level. The advantage of using Census data lies mainly in its coverage, virtually 100% complete, and sophisticated quality assurance processes aimed at minimising potential sources of errors such as undercounting, partial response, respondent error, and processing error [45]. In this study, analyses of the predictors of ACSCs were based on hospitalization data, using non-ACSCs as an approximation for those not hospitalized with an ACSCs. Future studies can be designed looking at predictors of ACSCs hospitalisation based on individual level data that includes patients that are hospitalised and those not hospitalised, although such data are hard to obtain. As the data are cross-sectional, the findings need to be interpreted with caution. The observed associations of socio-economic disadvantage, rurality and age with ACSCs admissions may not necessarily be causal. Although the Victorian ACSCs study has shown strong relationship of access with ACSCs , several other factors such as disease prevalence, co-morbidities, and physician practice patterns may also potentially explain observed differentials in this study [7,8]. These factors can be further explored in the form of case control or prospective studies that can compare ambulatory care provided to the patients from high and low socio-economic status before admissions to hospitals. Finally, repeated hospitalisations were not excluded from the analyses due to lack of a unique identifier, which may have biased our results if the predictor variables were associated with readmission rates across population groups.

## Conclusions

This study suggests that lack of timely and effective primary care may have significant impact on rates of admissions for ACSCs that vary by both individual- and area-level socio-demographics of the adult and paediatric population. Disadvantaged paediatric and adult population experience more need of hospital care for ACSCs. Access barriers to primary care are plausible causes for the observed disparities. These findings may have important implications for design and implementation of interventions for improving access to primary health care and reducing disparities in health.

## Abbreviations

ABS: Australian bureau of statistics; ACSCs: Ambulatory care sensitive conditions; ARIA: Accessibility/Remoteness index of Australia; ASGC: Australian standard geographic classification; CI: Confidence intervals; COPD: Chronic obstructive pulmonary disease; IRSED: Index of Socio-economic disadvantage; LGA: Local government areas; OR: Odds ratios; PCPs: Primary care partnerships; SEIFA: Socioeconomic indexes for areas; SES: Socio-economic status; VAED: Victorian admitted episodes dataset.

## Competing interests

The authors declare that they have no competing interest.

## Authors’ contributions

ZA conceived the study, including its design and coordination. SIH, HA and TdG assisted ZA in the statistical analysis and interpretation of the data. SIH and HA further assisted ZA in literature review and drafting of the manuscript. TdG and CS contributed to data interpretation, editing and critically revising the manuscript. All authors read and approved the final manuscript.

## Ethics statement

VAED administrative database is used in this paper. No human subjects or research on human subject was conducted. The data used are de-identified and only aggregate level information is presented. In addition, we i.e. the Health Intelligence Unit (HIU) are the secondary data custodians of the VAED.

## Pre-publication history

The pre-publication history for this paper can be accessed here:

http://www.biomedcentral.com/1472-6963/12/475/prepub
